# Drugmonizome and Drugmonizome-ML: integration and abstraction of small molecule attributes for drug enrichment analysis and machine learning

**DOI:** 10.1093/database/baab017

**Published:** 2021-03-31

**Authors:** Eryk Kropiwnicki, John E Evangelista, Daniel J Stein, Daniel J B Clarke, Alexander Lachmann, Maxim V Kuleshov, Minji Jeon, Kathleen M Jagodnik, Avi Ma’ayan

**Affiliations:** Department of Pharmacological Sciences; Mount Sinai Center for Bioinformatics; Big Data to Knowledge, Library of Integrated Network-Based Cellular Signatures, Data Coordination and Integration Center (BD2K-LINCS DCIC); Knowledge Management Center for Illuminating the Druggable Genome (KMC-IDG); Icahn School of Medicine at Mount Sinai, 1 Gustave L. Levy Place, Box 1603, New York, NY 10029, USA; Department of Pharmacological Sciences; Mount Sinai Center for Bioinformatics; Big Data to Knowledge, Library of Integrated Network-Based Cellular Signatures, Data Coordination and Integration Center (BD2K-LINCS DCIC); Knowledge Management Center for Illuminating the Druggable Genome (KMC-IDG); Icahn School of Medicine at Mount Sinai, 1 Gustave L. Levy Place, Box 1603, New York, NY 10029, USA; Department of Pharmacological Sciences; Mount Sinai Center for Bioinformatics; Big Data to Knowledge, Library of Integrated Network-Based Cellular Signatures, Data Coordination and Integration Center (BD2K-LINCS DCIC); Knowledge Management Center for Illuminating the Druggable Genome (KMC-IDG); Icahn School of Medicine at Mount Sinai, 1 Gustave L. Levy Place, Box 1603, New York, NY 10029, USA; Department of Pharmacological Sciences; Mount Sinai Center for Bioinformatics; Big Data to Knowledge, Library of Integrated Network-Based Cellular Signatures, Data Coordination and Integration Center (BD2K-LINCS DCIC); Knowledge Management Center for Illuminating the Druggable Genome (KMC-IDG); Icahn School of Medicine at Mount Sinai, 1 Gustave L. Levy Place, Box 1603, New York, NY 10029, USA; Department of Pharmacological Sciences; Mount Sinai Center for Bioinformatics; Big Data to Knowledge, Library of Integrated Network-Based Cellular Signatures, Data Coordination and Integration Center (BD2K-LINCS DCIC); Knowledge Management Center for Illuminating the Druggable Genome (KMC-IDG); Icahn School of Medicine at Mount Sinai, 1 Gustave L. Levy Place, Box 1603, New York, NY 10029, USA; Department of Pharmacological Sciences; Mount Sinai Center for Bioinformatics; Big Data to Knowledge, Library of Integrated Network-Based Cellular Signatures, Data Coordination and Integration Center (BD2K-LINCS DCIC); Knowledge Management Center for Illuminating the Druggable Genome (KMC-IDG); Icahn School of Medicine at Mount Sinai, 1 Gustave L. Levy Place, Box 1603, New York, NY 10029, USA; Department of Pharmacological Sciences; Mount Sinai Center for Bioinformatics; Big Data to Knowledge, Library of Integrated Network-Based Cellular Signatures, Data Coordination and Integration Center (BD2K-LINCS DCIC); Knowledge Management Center for Illuminating the Druggable Genome (KMC-IDG); Icahn School of Medicine at Mount Sinai, 1 Gustave L. Levy Place, Box 1603, New York, NY 10029, USA; Department of Pharmacological Sciences; Mount Sinai Center for Bioinformatics; Big Data to Knowledge, Library of Integrated Network-Based Cellular Signatures, Data Coordination and Integration Center (BD2K-LINCS DCIC); Knowledge Management Center for Illuminating the Druggable Genome (KMC-IDG); Icahn School of Medicine at Mount Sinai, 1 Gustave L. Levy Place, Box 1603, New York, NY 10029, USA; Department of Pharmacological Sciences; Mount Sinai Center for Bioinformatics; Big Data to Knowledge, Library of Integrated Network-Based Cellular Signatures, Data Coordination and Integration Center (BD2K-LINCS DCIC); Knowledge Management Center for Illuminating the Druggable Genome (KMC-IDG); Icahn School of Medicine at Mount Sinai, 1 Gustave L. Levy Place, Box 1603, New York, NY 10029, USA

## Abstract

Understanding the underlying molecular and structural similarities between seemingly heterogeneous sets of drugs can aid in identifying drug repurposing opportunities and assist in the discovery of novel properties of preclinical small molecules. A wealth of information about drug and small molecule structure, targets, indications and side effects; induced gene expression signatures; and other attributes are publicly available through web-based tools, databases and repositories. By processing, abstracting and aggregating information from these resources into drug set libraries, knowledge about novel properties of drugs and small molecules can be systematically imputed with machine learning. In addition, drug set libraries can be used as the underlying database for drug set enrichment analysis. Here, we present Drugmonizome, a database with a search engine for querying annotated sets of drugs and small molecules for performing drug set enrichment analysis. Utilizing the data within Drugmonizome, we also developed Drugmonizome-ML. Drugmonizome-ML enables users to construct customized machine learning pipelines using the drug set libraries from Drugmonizome. To demonstrate the utility of Drugmonizome, drug sets from 12 independent SARS-CoV-2 *in vitro* screens were subjected to consensus enrichment analysis. Despite the low overlap among these 12 independent *in vitro* screens, we identified common biological processes critical for blocking viral replication. To demonstrate Drugmonizome-ML, we constructed a machine learning pipeline to predict whether approved and preclinical drugs may induce peripheral neuropathy as a potential side effect. Overall, the Drugmonizome and Drugmonizome-ML resources provide rich and diverse knowledge about drugs and small molecules for direct systems pharmacology applications.

**Database URL**: https://maayanlab.cloud/drugmonizome/.

## Introduction

Currently, drug discovery efforts suffer from high attrition rates, long research and development timelines, and high financial costs (1, 2). Big Data applications to drug discovery include *in silico* docking drug screens, network-based and transcriptomics-based methods, as well as the combination of *in vitro* screens with computational predictions (3, 4). Drug repurposing is a strategy for elucidating novel indications for previously approved compounds with known safety profiles. This approach significantly mitigates the conventional drug discovery life cycle (5, 6). The process of drug repurposing usually involves the high-throughput screening of a library of approved and preclinical compounds to observe a particular desired phenotype. Such screens identify and prioritize potential therapeutic leads. The identified lead compounds may be a heterogeneous group of small molecules whose common mechanisms of action are unclear. *In vitro* screening techniques can be supplemented with computational methods to further investigate the connectedness among the top small molecule hits.

At the same time, gene set enrichment analysis (7) is a popular statistical method that computes significant overlap between an input gene set and libraries of annotated gene sets. Several online tools such as Enrichr (8, 9), WebGestalt (10) and DAVID (11) have used this paradigm to enable users to better understand their results from genomics, transcriptomics, epigenomics, proteomics and other omics. Enrichment analysis can be applied to drug and small molecule sets in a similar way. For example, drug set enrichment analysis was applied to analyze drug-induced gene expression profiles of small molecules that shared a phenotype of interest (12). Huang *et al.* expanded on the idea of drug set enrichment analysis by developing a tool called DrugPattern (13). DrugPattern analyzes drug sets, where a set of drugs is grouped under a common biomedical term. DrugPattern was demonstrated to predict drugs that may downregulate oxidized low-density lipoprotein, a molecule associated with the development of coronary heart disease. Predictions for novel compounds were confirmed *in vitro*. These two previous efforts to develop drug set enrichment analysis tools establish a good foundation for such analyses. However, these resources suffer from low coverage of unique small molecules and their associated biomedical attributes, as well as outdated web-based platforms that are not intuitive to use.

Here, we expand on previous drug set enrichment analysis efforts with Drugmonizome and Drugmonizome-ML. Drugmonizome is a database with a web-based interface for querying sets of small molecules and drugs to retrieve enriched biomedical terms. In contrast to prior tools, the drug set libraries within Drugmonizome are extracted from many more resources. In addition, the user interface of Drugmonizome provides fast enrichment analysis calculation, complex metadata queries and interactive visualization of the enrichment results, among other advanced features. Drugmonizome-ML is an interactive machine learning pipeline that is a counterpart to Drugmonizome. Drugmonizome-ML provides users with flexible options for creating customized machine learning models to predict novel attributes for small molecules and drugs, for example, side effects or indications.

The utility of Drugmonizome and Drugmonizome-ML is demonstrated via two case studies. To showcase the capabilities of Drugmonizome, we performed meta-analysis of 12 published *in vitro* drug screens to identify consensus features of compounds found to be effective against the coronavirus SARS-CoV-2. A case study that utilizes Drugmonizome-ML predicts whether preclinical small-molecule compounds and approved drugs will induce peripheral neuropathy as a side effect, based on transcriptomics and compound structural features.

## Materials and methods

### Harmonizing small molecule names and identifiers

Due to the inherent inconsistencies in the way small molecules and drugs are cataloged across various online repositories (14, 15), resolving unique small molecule entities among these resources required a standardized lexicon of small molecule names and synonyms. Previous efforts used the UniChem connectivity search (16) to map International Union of Pure and Applied Chemistry Chemical Identifier (InChI) key representations of small molecules from DrugBank (17) to unique identifiers from a variety of drug cataloging resources (18). The InChIKey is a widely used text-based identifier system for chemicals. The DrugBank database currently includes over 12 000 well-studied approved drugs and experimental small molecules that are annotated with a variety of metadata (17). Therefore, identifiers from popular chemical cataloging resources such as PubChem (19) and PharmGKB (20) could be cross-referenced with DrugBank to harmonize and standardize small molecule names and synonyms. This same methodology was adapted for the 2019 version of DrugBank. For this project, we created a master metadata table of small molecules and their associated identifiers. This includes synonymous names; InChIKeys; canonical simplified molecular-input line-entry system (SMILES) strings, an ASCII representation of small molecule structure; and resource-specific identifiers from DrugBank. In addition, experimental small molecules that were unique to the library of network-based cellular signatures (LINCS) project (21) were included in the master metadata table with their resource-specific identifiers. If any of these small molecule identifiers were not cataloged in DrugBank or the LINCS Common Fund program, we queried PubChem with the power user gateway-representational state transfer (PUG-REST) (22) application programming interface (API) (23) to retrieve the missing small molecule metadata. In addition, experimental small molecules that were unique to the LINCS project (21) were included in the master metadata table with their resource-specific identifiers.

### Creating the drug set libraries

Drug set libraries associate biomedical terms with drugs and small molecules. Drug set libraries are stored as drug matrix transposed (.DMT) files, a tab delimited file format that describes a collection of term–drug set associations. The 34 Drugmonizome drug set libraries contain drug–term associations collected from various online tools and repositories. We required that each set of drugs must include at least five small molecules. This requirement is to satisfy the minimum requirement for contingency table statistics with the Fisher’s exact test (24). Python scripts and Jupyter Notebooks were developed to process the data from each resource. These open-source pipelines generate the drug set libraries. Drug set libraries can be grouped into several categories that include (i) drug targets and associated genes; (ii) side effects, adverse events and phenotypes; (iii) gene ontology (GO) and pathway terms; (iv) chemical structure and sub-structure motifs; and (v) modes of action. Drug targets and drug–gene co-occurrences from literature were collected from several sources including (i) the Drug Repurposing Hub (25); (ii) DrugBank (17); (iii) DrugCentral (26); (iv) Harvard Medical School LINCS KINOMEScan (27) and (v) Geneshot (28). Drug-induced gene expression signatures were extracted from (vi) L1000 fireworks display (L1000FWD) (29); (vii) CREEDS (30) and (viii) search tool for interactions of chemicals (STITCH) (15). Drug to single nucleotide variant associations were extracted and processed from PharmGKB (20). Side effect information was collected from (i) Side Effect Resource (SIDER) (31); (ii) predicted side effects from the side effect prediction (SEP)-L1000 (32); and predicted side effects were also curated from (iii) OFFSIDES (33). Gene ontology terms were extracted from the Gene Ontology (34), and pathway terms were extracted from KEGG (35). These terms were associated with unique small molecules based on gene expression profiles. Upregulated and downregulated gene sets for each small molecule were separately queried via the Enrichr API (8, 9). Term–drug pairs with a significant *q*-value (Benjamini–Hochberg correction, *P* < 0.01) were included in the drug set library. Small molecules were grouped under their common upregulated or downregulated GO or pathway terms. Mechanisms of action and clinical indications for drugs were collected from (i) World Health Organization Anatomical Therapeutic Chemical (ATC) codes (36); (ii) The Drug Repurposing Hub (25) and (iii) SIDER (31). Finally, we grouped the drugs and small molecules by their shared structural features. As described above, a master list of every unique small molecule, and its metadata, retrieved across all resources was created. This master list included SMILES. RDKit is an open-source cheminformatics package capable of decomposing SMILES strings into descriptive bit vectors that describe the molecular features of a small molecule (37). The SMILES string of each small molecule from the Drugmonizome master list was converted into a bit vector array using the 166-bit Molecular ACCess System (MACCS) key dictionary (38) and the 881-bit PubChem fingerprint dictionary. Small molecules sharing the same bits corresponding to a common structural feature were grouped into sets and converted into respective MACCS and PubChem fingerprint drug set libraries.

### The Drugmonizome user interface

The Drugmonizome web-based application is built on an instance of the Signature Commons (https://github.com/MaayanLab/signature-commons). The Signature Commons software architecture is a skeleton general-purpose cataloging system with signature search capabilities. The Signature Commons database employs a hierarchical cross-referencing system that relies on universally unique identifiers attached to each unique resource, drug set library, drug set within each library, and small molecule entity within each of the drug sets. The front page includes a metadata search, where users can submit queries to retrieve information about single drugs and any search term found within descriptions of resources, libraries, drug sets and small molecules. The drug set enrichment analysis page enables users to submit a set of small molecule entities for enrichment analysis. These entities need to be entered as one entity in each row and can be a drug name, an InChIKey, a DrugBank ID, a Broad Institute (BRD) identifier or a SMILES string, depending on the level of specificity the user requires for the search. Entities within the Drugmonizome database may share the same name, although their stereochemistry may differ, as denoted by their associated InChIKey. If users are concerned with stereochemistry, they may opt to submit their queries as DrugBank ID, BRD-ID or InChIKey. Once the entity list is submitted, a results page is generated with identified enriched drug sets across all resources. Users can expand each resource to view the enrichment results from each drug set library. The specific enriched drug sets and overlapping small molecule entities are displayed in bar charts, volcano plots and interactive sortable tables. The resources page includes all the tools, databases and repositories from which the Drugmonizome data were compiled. Clicking on any of the resource cards directs users to a page that describes the resource. The ‘Tutorial’ and ‘API’ tabs include documentation for using the Drugmonizome website and API. Lastly, the ‘About’ page includes a variety of global statistics that visualize the coverage of biomedical terms and drug–term associations in Drugmonizome, including pie charts that visualize the relative contributions of each resource to the overall database.

### Computing drug set enrichment

The Fisher’s exact test (24) is the core method used to calculate the significance of overlap between two drug sets. It calculates the probability of observing overlap between two independent sets based on the hypergeometric distribution. Drugmonizome utilizes an implementation of the Fisher’s exact test that is optimized for speed. The enrichment analysis component, accessible via an API, is implemented as an independent Java servlet running on a Dockerized Apache Tomcat server.

### Creating the Drugmonizome-ML Appyter

Appyters are self-contained web-based bioinformatics applications that are created directly from Jupyter Notebooks (39). By inserting Jinja syntax into a Jupyter Notebook, the notebook becomes a template. This template is compiled into a full-stack Dockerized web-based application that presents the user with an HTML form that collects global variables needed for the notebook execution. Once the user fills the form and clicks submit, the notebook is executed in the cloud and the user is presented with the rendered executed notebook. The Drugmonizome-ML Appyter is an interactive web-based bioinformatics application built on top of the Drugmonizome database. The Drugmonizome-ML Appyter input form is composed of three sections: input dataset selection, target label selection and settings for the machine learning pipelines. Input features include all the drug set libraries included in Drugmonizome, as well as other datasets. Specifically, the Drugmonizome-ML Appyter includes features extracted from SEP-L1000 (32). These features include L1000 gene expression signatures (40), cell morphological features (41) and chemical fingerprints. The target label selection provides users with the ability to specify the target vector for predictions such as side effects, drug targets and indications. An autocomplete input field provides the ability to fetch a target vector from existing Drugmonizome drug sets. Optionally, users can upload a custom list of drugs with a common phenotype as the target binary target vector for classification. Lastly, the Drugmonizome-ML Appyter machine learning pipeline includes several scikit-learn (42) options for data normalization, dimensionality reduction, feature selection, classification algorithms and methods to evaluate the classifier. Once the input form is filled, a Jupyter Notebook is launched in the cloud with all user-selected settings, a model is trained and then the trained model is used to make predictions. After a job is completed, the results are stored in the cloud and can be shared via a unique URL that provides access to the executed Appyter notebook.

### Predicting peripheral neuropathy as a side effect using Drugmonizome-ML

A set of 19 898 compounds with L1000 gene expression features for 978 landmark genes were downloaded and processed from SEP-L1000 (32), and Morgan chemical fingerprints (radius = 4, nbits = 2048) were computed for each compound with RDKit. The binary Morgan fingerprint features were TF-IDF normalized to normalize for the frequency of different chemical structures. Out of the 19 898 compounds present within the input dataset, 226 drugs known to have the side effect ‘peripheral neuropathy’ were identified within the SIDER side effects drug set library and used as the positive class to make predictions for additional compounds that may cause this side effect based on shared properties with the positive-label compounds. The semantic mapping of small molecules between the SEP-L1000 and Drugmonizome drug set libraries was performed by matching the complete InChIKeys. To optimize the learning algorithm and hyperparameters, we used the scikit-learn Grid Search with 10-fold cross-validation and evaluated the Logistic Regression, Support Vector Machine, Extra Trees (ET) and Random Forest classifiers based on the Area Under the Receiver Operating Characteristic Curve (AUROC) and Area Under the Precision-Recall Curve (AUPRC) methods. Class weights were set to the inverse of class frequency to handle the class imbalance present within the input dataset. After model selection, we trained the best-performing ET model using 10-fold stratified cross-validation with three repeats. We then examined the validation-set predictions for each compound to identify additional compounds that were not known to induce peripheral neuropathy before but received high prediction scores.

## Results

### Drugmonizome database

**Figure 1.**
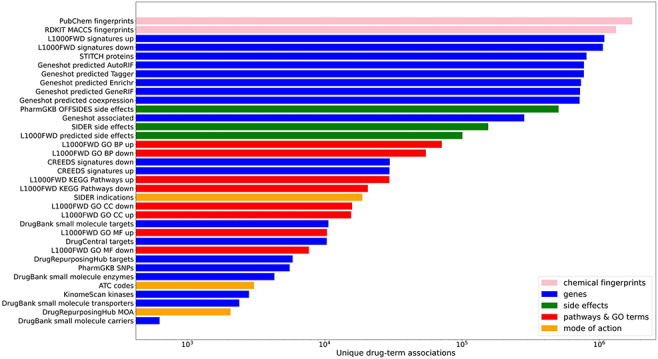
Counts of unique drug–term associations for each library. Terms are colored by their term type groupings.

**Figure 2.**
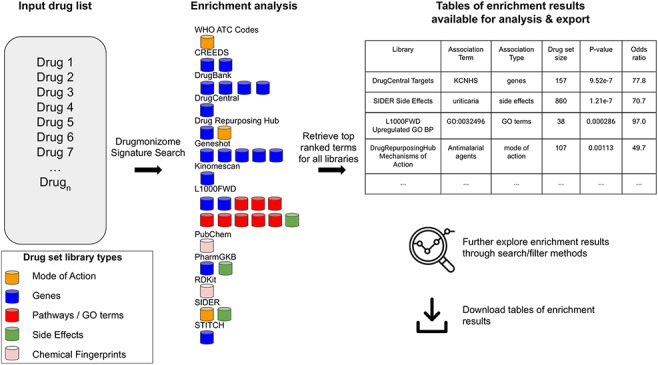
The Drugmonizome signature search workflow. A set of drugs is submitted for enrichment analysis across all the Drugmonizome gene set libraries. The enrichment results are provided in tables that enable further exploration of the overlapping drugs.

In total, small molecule data from 13 unique resources were transformed into 35 drug set libraries with a total of 10 395 794 drug–attribute associations organized into 110 903 drug sets spanning a variety of biomedical association terms (Table 1, Figure 1). 14 579 unique drugs and small molecules from DrugBank (17) and the LINCS project (21) are included in the Drugmonizome database. The Drugmonizome website includes a metadata search engine that enables users to input any search term. The returned results include matching drug sets, drugs, small molecules and other relevant entities. Information about drugs and small molecules can be accessed from landing pages for each small molecule or drug. These landing pages include a listing of all drug sets that contain the small molecule. Information about each drug set includes drug set size and the resource which the drug set was derived from. Additionally, the user can explore which small molecules are included in each matching set. The drug set enrichment analysis input form enables users to submit their own list of small molecules for enrichment analysis (Figure 2). Users can input small molecule lists by name, InChIKey, SMILES string and resource-specific identifiers such as those from DrugBank or the Broad Institute for LINCS small molecules IDs. A results page is generated for each drug set library where drug sets from each library are ranked based on overlap with the input drug set based on the Fisher’s exact test. The results from each library can be further examined by looking at all metadata associated with the enriched term. The drug set enrichment analysis can also be accessed programmatically using the Drugmonizome documented OpenAPI (43). Drugmonizome also has a resources tab that lists information about the 13 unique resources with links, PubMed IDs and other identifying resource-level metadata.

**Table 1.** List of drug set libraries served by Drugmonizome

Resource	Dataset	Drugs	Attributes	Average drugs per term
Geneshot	Tagger Predicted Genes	3938	13 882	55.60
Geneshot	Enrichr Predicted Genes	3938	11 845	62.03
Geneshot	AutoRIF Predicted Genes	3938	11 695	66.03
Geneshot	GeneRIF Predicted Genes	3938	9193	78.65
Geneshot	Coexpression Predicted Genes	3938	9087	78.95
STITCH	Targets_500	7303	9063	89.05
L1000FWD	Downregulated Genes	4884	7622	139.10
L1000FWD	Upregulated Genes	4884	7611	142.88
Geneshot	Literature Associated Genes	3938	7503	37.80
PharmGKB	Predicted Side Effects	1435	7137	70.72
CREEDS	Upregulated Genes	71	2535	11.67
CREEDS	Downregulated Genes	72	2532	11.76
SIDER	Side Effects	1635	2078	74.60
L1000FWD	Upregulated GO Biological Processes	4195	1228	58.03
L1000FWD	Downregulated GO Biological Processes	4013	1068	51.05
L1000FWD	Predicted Side Effects	4852	1013	99.34
SIDER	Indications	1546	867	21.66
PubChem	PubChem Fingerprints	13 379	669	2594.72
DrugBank	Drug Targets	4467	611	17.42
PharmGKB	Single Nucleotide Polymorphisms	483	554	10.02
DrugCentral	Genes	1555	540	19.16
DrugRepurposingHub	Genes	1720	375	15.57
ATC	ATC Codes	2233	308	9.91
KINOMEscan	Kinases	54	301	9.33
L1000FWD	Upregulated KEGG Pathways	3662	245	120.58
L1000FWD	Downregulated KEGG Pathways	3309	236	87.29
L1000FWD	Upregulated GO Molecular Function	2427	183	56.77
RDKit	MACCS Fingerprints	14 308	163	4080.18
L1000FWD	Downregulated GO Molecular Function	2158	158	48.56
L1000FWD	Downregulated GO Cellular Component	3246	157	100.82
DrugRepurposingHub	Mechanisms of Action	1854	154	13.37
L1000FWD	Upregulated GO Cellular Component	3366	153	101.87
DrugBank	Enzymes	1473	72	59.73
DrugBank	Transporters	832	51	46.80
DrugBank	Carriers	458	14	44.78

### Drugmonizome COVID-19 case study

**Figure 3.**
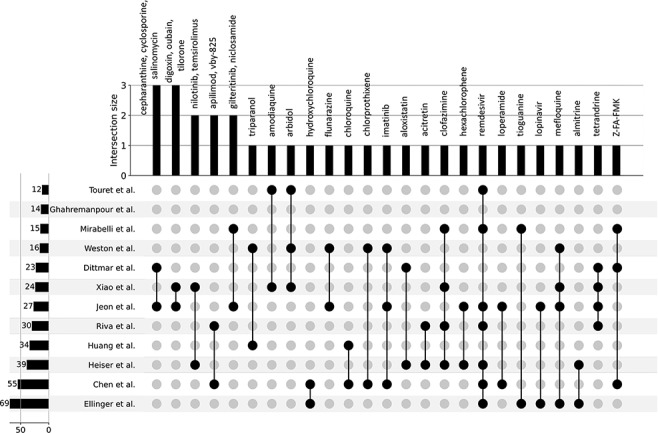
UpSet plot detailing the overlap among drug hits across 12 independent published *in*  *vitro* drug screen studies.

**Figure 4.**
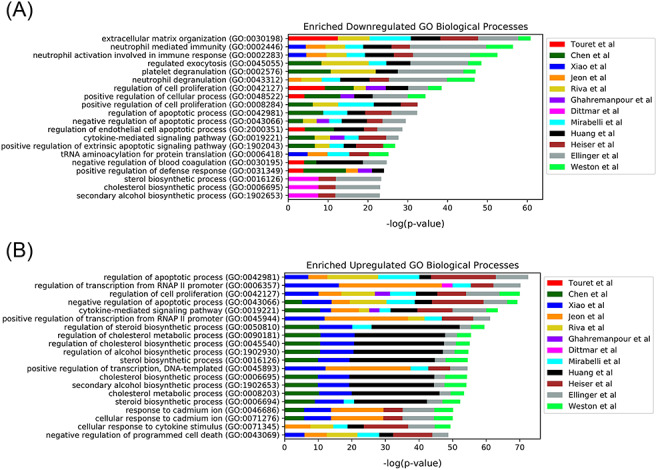
Top 20 enriched GO Biological Processes terms for the 12 *in*  *vitro* SARS-CoV-2 drug screens. Enriched terms are ranked by the sum of the −log(*P*-value) of the term across all screens. The enriched terms are applied to the consensus downregulated (A) and upregulated (B) genes for each drug in each set based on the data provided from L1000FWD (29).

In late 2019, the novel coronavirus, SARS-CoV-2, emerged in China and has since claimed many lives and caused widespread economic disruption (44, 45). Countless research groups in the scientific community refocused their efforts toward discovering therapeutics for COVID-19. Given the immense resources required for developing and testing novel small molecules, many groups turned to drug repurposing—an alternative avenue for expedited discovery of therapeutics with known safety profiles. The COVID-19 Drug and Gene Set Library (46) was developed to collect drug and gene sets related to COVID-19, including drug sets extracted from 12 publications that describe SARS-CoV-2 *in vitro* drug screens (47–58). While there is not much overlap among the hits from the 12 independent *in vitro* drug screens (Figure 3), these drug sets share the common phenotype of inhibiting SARS-CoV-2 infection in cell-based assays. Drugs and small molecules were predominantly cataloged by name. Therefore, these entities could only be resolved by their common name because identifiers were not supplied in most cases (Supplementary Table S1). The drug sets from these *in vitro* screens were independently submitted to Drugmonizome for enrichment analysis to highlight potential common themes across the screening results. To determine commonalities among the drug hits in perturbing the same biological processes, the top enriched terms from the up- and downregulated L1000FWD GO Biological Processes drug set libraries were collated. The top 20 terms across the enrichment results were determined by the largest cumulative −log *P*-values, and the contribution to the total by each drug screen was visualized as stacked bar plots (Figure 4). Notably, among the pooled enrichment results for the 12 *in vitro* drug screens hits there was a common theme of upregulated terms related to cholesterol metabolism, including regulation of cholesterol metabolic process (GO:0090181), regulation of cholesterol biosynthetic process (GO:0045540), sterol biosynthetic process (GO:0016126) and cholesterol biosynthetic process (GO:0006695). It was recently demonstrated that drugs that upregulate the genes related to cholesterol biosynthesis can block SARS-CoV-2 in human cell lines and organoids (59).

### Drugmonizome ETL scripts, consensus analysis and machine learning Appyters

Appyters are bioinformatics web-based applications created from Jupyter Notebooks (39). By placing special code inside a standard Jupyter Notebook, and compiling the notebook with the Appyter SDK, the notebook is converted into a fully functional web application. The Appyter web-based application first presents the user with an input form, where they can upload files and submit input parameters. When submitted, the Jupyter Notebook is executed in the cloud and a report is generated and presented to the user. Users are also provided with a permanent link to the executed notebook, options to download the notebook, download the output from the notebook and apply further customization to the results. The Appyters Catalog provides a collection of Appyters developed by the community. The Drugmonizome extracting, transforming and loading (ETL) Appyters are a collection of Appyters that convert data from various online resources that provide knowledge about drugs and small molecules into drug set libraries for Drugmonizome. Hence, several Appyters for ETL data from each resource in Drugmonizome were created for the purpose of automating the process of updating all drug set libraries. The Jupyter Notebooks used to create these Appyters are openly shared and versioned on GitHub. This approach provides simple mechanisms to continually update the Drugmonizome resource. The Drugmonizome Consensus Appyter streamlines the analysis of a collection of drug sets. After uploading a file containing drug sets, users can select the Drugmonizome drug set libraries for enrichment analysis, as well as how many top consensus terms to visualize. When executed, the Appyter produces a report that contains a stacked bar chart with the cumulative ranks of enriched terms from each library. An example is the chart provided for the SARS-CoV-2 *in vitro* drug screens case study (Figure 4). The Appyter also produces downloadable tables and heatmaps.

### Drugmonizome-ML Appyter and the peripheral neuropathy case study

**Figure 5.**
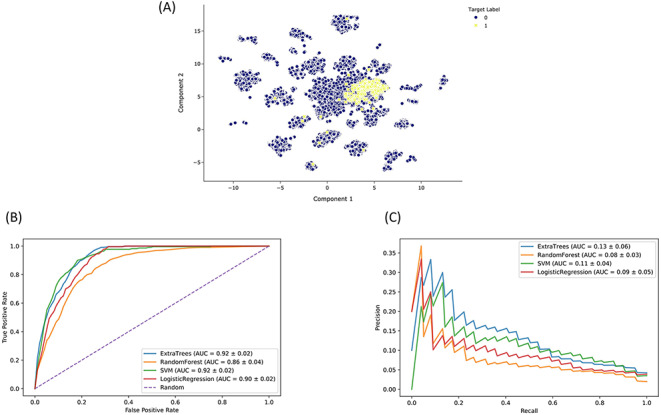
Drugmonizome-ML classifier for prioritizing drugs that may induce peripheral neuropathy. (A) Input feature space with Uniform Manifold Approximation and Projection (UMAP) dimensionality reduction. Each point represents one of 19 898 compounds with 3026 features per compound. Compounds with the known side effect of peripheral neuropathy are highlighted in yellow. (B) ROC and (C) PRC across cross-validation splits after hyperparameter optimization for each classifier to predict peripheral neuropathy. Each curve shows the mean ROC and standard deviation after 10-fold cross-validation for each classifier.

The Drugmonizome-ML Appyter is a customizable machine learning pipeline that is available as an Appyter. Using an HTML input form, Drugmonizome-ML enables users to choose feature matrices and target vectors to construct machine learning tasks for predicting drug attributes. The user has the option to choose from various scikit-learn (42) settings to customize and evaluate a user-selected classifier algorithm. As a case study, we trained a classifier to identify preclinical and approved drugs that may cause peripheral neuropathy as a side effect. Peripheral neuropathy is a debilitating side effect for many drugs, common among chemotherapeutics (60). It causes loss of sensation or pain in the hands and feet, as well as overall weakness and pain. Peripheral neuropathy is also a side effect of diabetes (61). Since many critical side effects may be missed during clinical trials, computationally predicting side effects such as peripheral neuropathy for new drug applications can alert physicians about potential side effects to watch for during clinical trials. A collection of 19 898 compounds characterized by their effects on gene expression and their chemical fingerprint features were used to train and evaluate a classifier that can predict associations between compounds and peripheral neuropathy. The input dataset for constructing the classifier consisted of L1000 gene expression signatures of 978 landmark genes after perturbation with each compound (32, 40) and Morgan fingerprints (radius = 4, nbits = 2048) generated with RDKit (37). Compounds known to cause peripheral neuropathy were curated from SIDER (31). We evaluated various classifier algorithms after hyperparameter optimization based on AUROC and AUPRC (Figure 5). Based on this analysis, we selected the ET classier due to its short training time and marginally better AUPRC. We trained an ET classifier (n_estimators = 1250, class_weight = balanced, max_features = log2, criterion = entropy) with 10-fold cross-validation repeated three times to predict novel compounds that may cause peripheral neuropathy as a side effect. The top-ranked predicted compounds are ranked by their mean prediction probabilities (Tables 2 and 3).

**Table 2.** Top 15 drugs predicted by the ET model that are known to be associated with peripheral neuropathy from SIDER

InChIKey	Name	Known	Prediction probability
JURKNVYFZMSNLP-UHFFFAOYSA-N	Cyclobenzaprine (BRD-K42348709)	TRUE	0.8592
KRMDCWKBEZIMAB-UHFFFAOYSA-N	Amitriptyline (BRD-K53737926)	TRUE	0.8311
MJIHNNLFOKEZEW-UHFFFAOYSA-N	Lansoprazole (BRD-A49172652)	TRUE	0.7613
ZZVUWRFHKOJYTH-UHFFFAOYSA-N	Diphenhydramine (BRD-K47278471)	TRUE	0.7153
ZKMNUMMKYBVTFN-HNNXBMFYSA-N	Ropivacaine (BRD-K50938786)	TRUE	0.582
BCGWQEUPMDMJNV-UHFFFAOYSA-N	Imipramine (BRD-K38436528)	TRUE	0.5591
WUBBRNOQWQTFEX-UHFFFAOYSA-N	Aminosalicylic acid (BRD-K80267133)	TRUE	0.4977
YREYEVIYCVEVJK-UHFFFAOYSA-N	Rabeprazole (BRD-A39390670)	TRUE	0.457
PHTUQLWOUWZIMZ-GZTJUZNOSA-N	Dosulepin (BRD-K54759182)	TRUE	0.3622
XRECTZIEBJDKEO-UHFFFAOYSA-N	Flucytosine (BRD-K82143716)	TRUE	0.3463
ODQWQRRAPPTVAG-BOPFTXTBSA-N	Doxepin (BRD-K37694030)	TRUE	0.3403
UGJMXCAKCUNAIE-UHFFFAOYSA-N	Gabapentin (BRD-K62737565)	TRUE	0.333
KBOPZPXVLCULAV-UHFFFAOYSA-N	Mesalazine (BRD-K28849549)	TRUE	0.3244
GBXSMTUPTTWBMN-XIRDDKMYSA-N	Enalapril (BRD-K57545991)	TRUE	0.3153
HCYAFALTSJYZDH-UHFFFAOYSA-N	Desipramine (BRD-K60762818)	TRUE	0.3102

**Table 3.** Top 15 drugs predicted by the ET model that are unknown to be associated with peripheral neuropathy

InChIKey	Name	Known	Prediction probability
NRUKOCRGYNPUPR-OQMCATNJSA-N	PLX-4720 (BRD-K16478699)	FALSE	0.9757
NRUKOCRGYNPUPR-OQMCATNJSA-N	Teniposide (BRD-A35588707)	FALSE	0.9396
STQGQHZAVUOBTE-INJOJONLSA-N	Daunorubicin (BRD-K91966436)	FALSE	0.8372
VSJKWCGYPAHWDS-FQEVSTJZSA-N	Camptothecin (BRD-K37890730)	FALSE	0.7782
FPIPGXGPPPQFEQ-OVSJKPMPSA-N	Retinol (BRD-K22429181)	FALSE	0.7499
LTMKESNXUBQKBP-UHFFFAOYSA-N	Lapatinib (BRD-M07438658)	FALSE	0.7442
HHJUWIANJFBDHT-KOTLKJBCSA-N	Vindesine (BRD-K59753975)	FALSE	0.7429
XECQQDXTQRYYBH-UHFFFAOYSA-N	Norcyclobenzaprine (BRD-K63165456)	FALSE	0.6919
FPIPGXGPPPQFEQ-UHFFFAOYSA-N	Tretinoin (BRD-K64634304)	FALSE	0.6753
XUBOMFCQGDBHNK-UHFFFAOYSA-N	Gatifloxacin (BRD-A74980173)	FALSE	0.6338
AJLFOPYRIVGYMJ-INTXDZFKSA-N	Mevastatin (BRD-K94441233)	FALSE	0.6235
KPQZUUQMTUIKBP-UHFFFAOYSA-N	Secnidazole (BRD-A70083328)	FALSE	0.5208
METKIMKYRPQLGS-LBPRGKRZSA-N	Atenolol (BRD-K44993696)	FALSE	0.4875
KGUMXGDKXYTTEY-FRCNGJHJSA-N	4-Hydroxyretinoic acid (BRD-A96799240)	FALSE	0.4861
BUJAGSGYPOAWEI-UHFFFAOYSA-N	Tocainide (BRD-A92670106)	FALSE	0.4753

## Discussion

The ability to perform drug set enrichment analyses for sets of small molecules against drug set libraries curated from public repositories and biomedical literature using the Drugmonizome web-based interface can shed light on the connectedness of sets of small molecule hits generated from drug screens. The COVID-19 case study highlighted a global theme that connects results from 12 independent *in*  *vitro* drug screens. Despite the minimal overlap among the hits across these screens, GO terms related to regulation of cholesterol metabolism and cell cycle were significantly enriched across the 12 independent drug sets. It should be noted that the cholesterol biosynthesis metabolic pathway is not just producing cholesterol, it is known to produce more than 300 metabolites. A few of these are likely critical to the virus life cycle. It has been reported that patients with high cholesterol and hypertension are at a higher risk of developing COVID-19 (62), and previous literature reports that cholesterol has important functions in regulating immune function, namely through alteration of plasma membrane cholesterol content, which may have effects on viral entry into cells (63, 64). Furthermore, several independent studies suggest that statins, which are cholesterol-lowering drugs, may reduce the severity of COVID-19 (65–68). While this evidence appears as a contradiction, lowering vs. increasing the level of cholesterol, it may be because the drugs that block the virus *in vitro* simply induce the expression of the cholesterol biosynthesis pathway and do not necessarily increase the production of cholesterol. Specifically, these drugs collectively upregulate the genes belonging to this pathway, while it was shown that the virus downregulates the same genes (59). Further understanding the exact metabolites that lead to increase or attenuation of infection requires further exploration. It should be noted that the drug sets used for this case study come from the COVID-19 Drug and Gene Set Library (46). This site provides links to drug set enrichment analysis with DrugEnrichr (69) (https://maayanlab.cloud/DrugEnrichr/). DrugEnrichr was developed by us to provide drug set enrichment analysis using the same drug set libraries created for Drugmonizome. This was achieved by simply swapping the Enrichr gene set libraries with the Drugmonizome drug set libraries. DrugEnrichr has fewer features when compared with Drugmonizome, for example, it does not have entity resolution, drug landing pages and extensive metadata search. The underlying database and enrichment analysis calculation in Drugmonizome and DrugEnrichr are identical. Hence, users may prefer the simpler user interface provided by DrugEnrichr. However, we recommend using Drugmonizome over DrugEnrichr.

For our second case study, we utilized the Drugmonizome-ML Appyter to make predictions and impute knowledge. Drugmonizome-ML provides researchers with the ability to construct custom machine learning pipelines using a simple input form. We used Drugmonizome-ML to predict peripheral neuropathy as a side effect for ∼20 000 preclinical and approved compounds. Among the top-ranked compounds that were not known to induce peripheral neuropathy from our input dataset were PLX-4720, a BRAF kinase inhibitor (70); camptothecin, a topoisomerase inhibitor (71); vindesine, a vinblastine derivative antineoplastic (72); and various forms of retinol, a fat-soluble vitamin (73). Additionally, stereoisomers of compounds known to induce peripheral neuropathy such as lapatinib, teniposide and daunorubicin were ranked as the top predicted compounds when left out as positives from the target prediction vector. This case study provides further evidence that Drugmonizome-ML can be used to prioritize compounds that induce peripheral neuropathy based on their transcriptomic profiles and chemical fingerprints. The top predicted compounds were predominantly chemotherapeutics that are enzyme inhibitors. It is well established that peripheral neuropathy is a common side effect among many therapeutics for cancer. Because clinical trials cannot capture all possible adverse effects of a therapeutic, computationally predicting compounds that may have severe side effects before they reach the market is vital for preventing unwanted consequences of treatment for patients. Beyond predicting side effects, Drugmonizome-ML provides the ability to predict other drug attributes. In fact, any attribute from the Drugmonizome drug set libraries such as indications, targets and others can be set up for constructing machine learning predictive models. Drugmonizome-ML targets researchers with no coding skills, but it is also expected to be useful for computationally savvy users that would utilize the Drugmonizome-ML framework as a skeleton for rapidly developing their ML models. It should be noted that the data within the Drugmonizome database is highly abstracted. This results in loss of information that may be critical to obtain optimal predictions. Regardless of such limitations, Drugmonizome and Drugmonizome-ML provide rich and well-organized knowledge about drugs and small molecules to facilitate and accelerate early-stage drug discovery efforts.

## Supplementary Material

baab017_Supp

## Data Availability

The Drugmonizome web site: https://maayanlab.cloud/drugmonizome The Drugmonizome-ML Appyter: https://appyters.maayanlab.cloud/#/Drugmonizome_ML The Drugmonizome ETL Appyters: https://appyters.maayanlab.cloud/#/?q=ETL%20&tags=Drugmonizome The Drugmonizome Consensus Appyter: https://appyters.maayanlab.cloud/#/Drugmonizome_Consensus_Terms Source code for the drug set library processing scripts: https://github.com/MaayanLab/Drugmonizome Source code for the ETL Appyters: https://github.com/MaayanLab/Drugmonizome-Data-Processing-Appyters Source code for the Drugmonizome Consensus Appyter: https://github.com/MaayanLab/appyter-catalog/tree/master/appyters/Drugmonizome_Consensus_Terms Source code for the Drugmonizome-ML Appyter: https://github.com/MaayanLab/Drugmonizome-ML
